# Stochastic Resonance Training Improves Balance and Musculoskeletal Well-Being in Office Workers: A Controlled Preventive Intervention Study

**DOI:** 10.1155/2018/5070536

**Published:** 2018-09-13

**Authors:** Yannik Faes, Clare Maguire, Michele Notari, Achim Elfering

**Affiliations:** ^1^Department of Work and Organizational Psychology, University of Bern, Bern, Switzerland; ^2^Department of Physiotherapy, BZG Bildungszentrum Gesundheit Basel-Stadt, Munchenstein, Switzerland; ^3^PHBern-School of Teacher Education, University of Applied Sciences, Bern, Switzerland

## Abstract

Sixty-two office workers in a Swiss federal department were randomly assigned to a training and a control group. While the training group was instructed to complete 3 stochastic resonance whole-body vibration (SR-WBV) exercises every week for 4 weeks, the control group received no treatment. During this time all participants answered a daily questionnaire concerning their surefootedness, sense of balance, musculoskeletal well-being, and muscle relaxation. Before and after the 4-week SR-WBV intervention, balance was tested with a single-leg stance on a foam mat of the Balance Error Scoring System (BESS) using a SwayStar™-System measuring Total Angle Area (TotAngArea) and Total Velocity Area (TotVelArea). Multilevel results highlighted a significant increase over time for surefootedness and sense of balance (t = 2.491, p = .016), as well as for musculoskeletal well-being and muscle relaxation (t = 2.538, p = .014) in the training group but not in the control group. Balance tests showed improvement of balance in the training group (TotAngArea: Z = 2.550, p = .011; TotVelArea: Z = 3.334, p = .001) but not in the control group. SR-WBV exercise indicated a high compliance during this study (3.87±0.45 trainings per week) underlining its benefits for the working context. Especially office workers who spend most of their time in sitting position could profit from SR-WBV exercise to improve balance and reduce the risk of falls.

## 1. Introduction

Every fourth accident in Switzerland is due to a slip, trip, and fall (STF) incident [1]. STF are also the most frequent accidents among office workers [2]. A 6-year longitudinal study about accidents in hospitals has shown that office workers and nurses have the most frequent STF incidents [3]. To prevent STF incidents risk factors must be identified [3]. On the one hand risk factors for STF of external origin are wet surfaces, poor lighting, lack of or inadequate handrails, and use of lifting aids [4]. Individual STF risk factors on the other hand are alcohol, smoking, inactivity, sleep disorders, and request for a job change [5]. Additional individual risk factors for STF are sex and age, whereas falls are more frequent in females and older individuals [6]. Also, balance has shown to decline with age and it was found that males have a better balance in more demanding balance tasks than females [7]. Because a loss of balance is a possible influence of individual frailties on STF [6], balance trainings are recommended to reduce STF [8, 9]. The World Health Organization (WHO) recommends in its physical activity strategy 2016-2025 more physical activity during the working day: “The measures could include action to address the workplace layout, such as the provision of adjustable desks, prominent and promotional signs on staircases encouraging their use, regular breaks during the day to allow for physical activity and membership of a gym or sports club, or, for larger companies, company-run sport facilities and programmes” [10].

A recent study has shown that a 13-week standardized exercise equipment-free program is effective in improving balance in the elderly [11]. In many worksite activity trainings participants have to invest more than 20 minutes for a training session [12] which results in a lack of compliance and a low participation rate [13, 14]. An easier way to implement more activity into the working day is through stochastic resonance whole-body vibration (SR-WBV). While evidence for long-lasting vibration frequency at work, as a risk factor for musculoskeletal diseases, is substantial [15], further research showed beneficial training effects of brief vibration experience [16, 17]. As described by Elfering et al. [18], “SR-WBV is low in nonmonetary effort when compared to conventional exercise: SR-WBV has very short exercising duration (about 10 minutes), is easily carried out in work settings, and no change of clothes is necessary.” Compliance rates with SR-WBV are higher than with other worksite activity programs, because it is less time consuming and can be performed easily and no physical exertion happens [19].

The outcome of SR-WBV at the worksite is promising. A 12-week course of low-frequency vibrating board therapy on patients with nonspecific low back pain showed improvements of balance of about 25% [20]. It has been shown that SR-WBV increases surefootedness and balance in health-care professionals [21] and white-collar employees [18]. Kaeding et al. [22] concentrated on the effects of sinusoidal WBV on balance comparing training and control group in office workers but did not find any improvement. In our study, we expect surefootedness and sense of balance to increase in those who are doing the SR-WBV program (H1). Additionally to these self-report measurements we expect those who complete the SR-WBV exercise to improve in an objective balance test (H2). Despite improvement of balance, SR-WBV reduced musculoskeletal disorders in an eight-week randomized-controlled trial on the employees of a university hospital, especially on those with baseline health restraints [13]. Also, four weeks of SR-WBV showed an increase of musculoskeletal well-being in the workers of a steel manufacturing company [23] as well as in employees engaged in sedentary work [18]. We expect office workers who are doing the 4-week SR-WBV intervention to experience an increase in musculoskeletal-wellbeing and muscle relaxation (H3). No effects are expected for the control group, in which participants are doing no SR-WBV exercise during this time.

### 1.1. How Stochastic Vibration Works

Vibration frequency at sinusoidal vibration is constant, whereas stochastic vibration changes randomly within a frequency range. Ward and colleagues [24] described stochastic resonance as “a nonlinear cooperative effect wherein the addition of a random process, or ‘noise' to a weak signal, or stimulus results in improved detectability or enhanced information content in some response.” The human body cannot foresee impending vibration movements during stochastic vibration and, therefore, is constantly challenged to adapt its neural and muscular reactions. Furthermore, no muscular fatigue is indicated by the human body during the application [25–28]. An interaction of different types of neurophysiologic sensors and the adjustment of afferent and efferent signals seems to be provoked by stochastic vibration, which may act as exercise for the sensorimotor system [25]

In the current longitudinal randomized-controlled trial we expect SR-WBV to improve surefootedness and sense of balance over time. The training group is expected to improve in a balance test. Additionally, we expect SR-WBV to improve musculoskeletal well-being and muscle relaxation.

## 2. Materials and Methods

### 2.1. Participants

Participants were office workers at a Swiss federal department. In team meetings all employees (N = 101) were informed about the study. The exclusion criteria were being pregnant and having osteosynthesis material (such as implants, screws) in the body, musculoskeletal disorders, joint problems (especially regarding the knee, hip, and back), herniated discs, rheumatism (such as spondylitis, gout, osteoporosis, and osteoarthritis), cardiovascular complaints, and disorders related to the sense of balance (such as a hearing loss). Nineteen employees (18.81%) could not participate because of the exclusion criteria, whereas 20 (19.80%) had other reasons (e.g., no time) not to participate. Sixty-two participants were randomly assigned to two groups containing 31 subjects each. Before the intervention started, five subjects had to be transferred from training into control group due to a business trip during the intervention time. All participants (32 male, 30 female) were between 18 and 63 (40.35 ± 14.17) years old. Forty-six participants were employed full-time, nine were working on an 80% time schedule (four days a week), four had a 50% time schedule (2.5 days a week), and one was working on a 90% time schedule (4.5 days a week). Fifty-one participants described their health status as rather good or very good and nine as moderate or rather bad. Eighteen participants did sports three to six times a week, 17 did sports at least once a week, 15 did sports only one to three times a month, and five did no sports. There were five people doing some type of sports every day. Ten participants were smokers and 50 were nonsmokers. Two participants did not answer the question about activity and smoking and one did not answer the question about their health status.  Table 1 depicts the descriptive study results for both groups. The training and control group did not differ in any variable at baseline.

### 2.2. Design and Procedure

The present study at a Swiss federal department was carried out as a health intervention for office workers. Therefore, all 101 office workers were informed about the goals of the present study and asked to participate. The study was presented in front of the top-management in July 2017. Thereby, participants were given the opportunity to complete every task regarding this study (SR-WBV exercises, answering of the questionnaires as well as the balance tests) within working hours. The study was then presented in front of the sub-teams, which consisted of about 10 people each. They had a test run with the stochastic resonance training (SRT) device and filled in the registration form where they were informed about their rights including to stop the training whenever they wanted to. Participants were given a guarantee of anonymity. All participants provided informed consent prior to their inclusion in the study. The study was performed in consensus with recommendations outlined by the Declaration of Helsinki and with all requirements defined by the Swiss Society of Psychology. The ethical committee of the responsible university faculty has approved the study proposal (Proposal No. Nr. 2017-08-00003).

This intervention study lasted four weeks and the design was a randomized-controlled trial. During this time the training group was instructed to train three times a week on a SRT-device, which was placed in a separate room at the entrance of the office-building. The control group received no treatment. They were not allowed to use the SRT-device until the study was completed. Participants of both groups were asked to answer a short daily questionnaire five days a week, from Monday to Friday. The daily questionnaire started one week before the SR-WBV exercises and stopped one week after. Before and after the 4-week SR-WBV intervention, balance was tested with a single-leg stance (on a foam mat) of the Balance Error Scoring System (BESS) [29] using a SwayStar System (Balance International Innovations GmbH, Switzerland).

### 2.3. Stochastic Resonance Whole-Body Vibration Training

**Figure 1:**
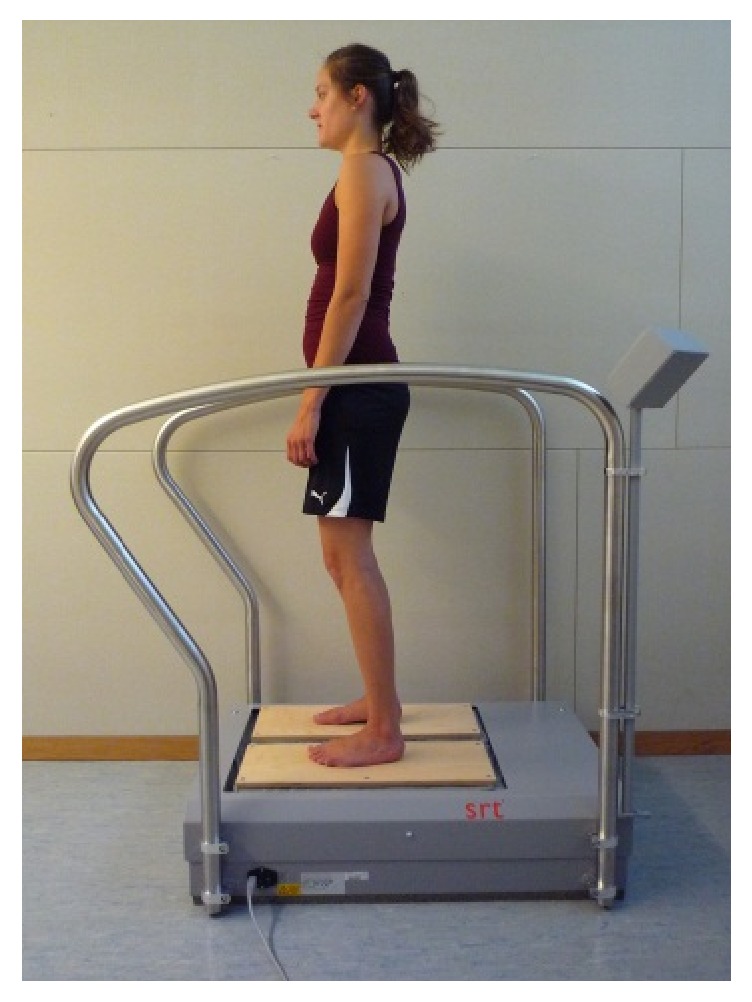
Starting position on SRT-device.

For the 4-week vibration training intervention a SRT-Zeptor Medical plus noise (FreiSwiss AG, Zurich, Switzerland) was used (Figure 1). It is made up of two independent, one-dimensional (up/down) stochastically oscillating footboards (3 mm amplitude), with two passive degrees of freedom (left/right, forward/backward). In addition to vertical and horizontal actions the platforms also allow medial and lateral tilting, which leads to a pluridimensional movement. Participants were instructed to stand on the footboards with their arms hanging loosely at their sides and with slightly bent knees (i.e., a skiing posture). One training session consisted of three series with a one-minute vibration training and a one-minute break in between. The vibration frequency was between 5 Hz and 6 Hz. Every participant received a personal instruction at the beginning. For organizational reasons, the SRT-device was installed on an Outlook Calendar and every participant was able to check the availability and book the SR-WBV sessions from their computer, just like a room reservation. Also, participants were encouraged to carry out the training with another participant, so that when one person was having the one-minute break, the other could exercise. Therefore, participants were free to train whenever they wanted three times a week. The training setting with three trainings a week was based more on empirical experience with other worksite SR-WBV studies [18, 23] than on scientific evidence, because the training parameters of SRT show a wide range of applications that are not as well known as they are for strength or endurance training [30].

Participants were only supervised once in a while and were advised when they had questions concerning the SR-WBV exercises. To date, only Kaeding et al. [22] chose an unsupervised intervention with WBV and reached a high compliance of 81.1%. Overall, participants completed a minimum of two and a maximum of five trainings every week (3.87±0.45 trainings per week). Therefore, participants even trained 29% more than required.

### 2.4. Daily Questionnaire

In a daily questionnaire participants were asked to answer two short questions about their balance:* “How do you rate your personal feelings about your balance today?”* and* “How sure-footed did you feel today?”* The answers on a 100-point rating-scale with 0 being* “a lot worse than usual*,” 100* “much better than usual*,” and 50* “same as always*.”

As the sample was relatively healthy, a floor-effect could be expected in relation to pain-related outcomes. Due to this assumption* musculoskeletal well-being *and* muscle relaxation* instead of musculoskeletal pain were assessed in the daily questionnaire, as was also done in previous studies [18, 23]. All scales were assessed in a 100-point-rating-scale with zero being* “not at all comfortable”* and 100* “as comfortable as you can imagine.”* The daily questionnaire was accessible via an internet-link to Qualtrics^©^ (2016 Qualtrics LLC). Participants were asked to fill in this questionnaire every morning from Monday to Friday before they started to work. In addition to these outcomes, demographical questions were asked at the beginning of the study.

### 2.5. Balance Test

Before and after the 4-week SR-WBV intervention, balance was tested with one stance (single-leg foam) of the Balance Error Scoring System (BESS) [29] using SwayStar (Balance International Innovations GmbH, Switzerland). BESS, which has been shown to correlate well with other measures of balance, can detect balance deficits in participants with concussion and fatigue. BESS scores increase with age and with ankle instability. Additionally, BESS scores are reported to improve after training [29]. Reliability of the BESS to assess static balance ranges from moderate (< 0.75) to good (> 0.75), although some authors report low levels of reliability [31]. Criterion-related validity is moderate to high, but the level of agreement depends on the testing condition. Difficult stances (e.g., single-leg foam,* r* = 0.79) have higher agreement compared to easier stances (double-leg foam,* r* = 0.31) [29].

Therefore, participants were instructed to stand on their dominant leg on a foam mat focusing on a point on the wall. Total angle area (TotAngArea) and total velocity area (TotVelArea) of trunk sway were measured during the stance. TotAngArea is defined by the envelope of the pitch and roll angular excursions over the complete trial. The unit is (deg)^2^. TotVelArea is defined by the envelope of the pitch and roll velocity excursions over the complete trial. The unit is (°/s)^2^. In both variables lower values stand for a better balance.

**Figure 2:**
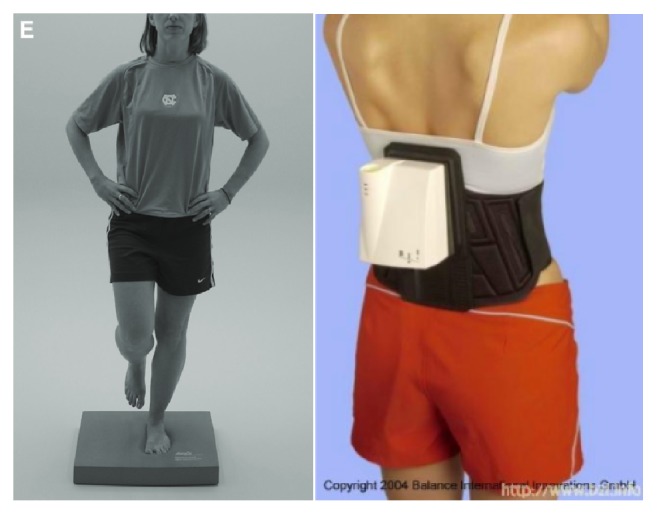
Dominant single-leg stance with foam of the Balance Scoring System (BESS) [29] was recorded with SwayStar (Balance International Innovations GmbH, Switzerland).

The recording of the trunk sway lasted three times twenty seconds with a 10-second pause between them. Similar to del Pozo-Cruz et al. [20] who stated that previous studies have reported similar results for the dominant and nondominant legs we focused on the dominant leg due to cost-considerations. The single-leg stance and the SwayStar System are shown in Figure 2.

### 2.6. Data Analysis

For the hypotheses 1 and 3 data was analyzed with longitudinal multilevel analysis [32] using the* MLwiN* software package version 3.00 [33]. The level of significance was p < .05 (two-tailed). Dependent variables were surefootedness, sense of balance, musculoskeletal well-being, and muscle relaxation with time (level 1) nested within persons (level 2). As outcomes were collected on a daily basis, time was represented in days [32]. Time range went from 0 (first day) to 28 (last day), with the* intercept* representing outcome status at the end of a period. A dummy variable represented the intervention (SR-WBV (1) vs. no treatment (0)).

Differences between as well as within participants were expected in the outcomes over time. Therefore, the* intercept* was conceptualized as random effect on both levels. Since overall effect of SR-WBV and difference in rate of change was of primary interest in the present study and data collection occasions were equal for all subjects in the sample (each day), time, and training effect over time as predictors were all set as fixed effects. Hence, the regression model assumed that the measured outcomes over time and dependence of training would be equal for all participants; i.e., no variation in individual regression slopes was postulated. The general model, which was used to test the improving effects of SR-WBV on the measured outcomes, contained only these three variables. It is represented by the following:

(1) outcomeij=β0ij constant+β1ij time+β2ij training+β3ij training×timeβ0ij=β0+u0j+e0ij

Subscript* i* indicates the level 1 (time) variable and* j* indicates the level 2 (person) variable. Outcomes were all mentioned above.

For hypothesis 2 data was analyzed with nonparametric sign test using SPSS software version 24 [34]. The sign test is a distribution-free test on the differences in trunk sway between individual pre- and postintervention levels.

## 3. Results

Data of sixty-two participants were analyzed. The training and control groups did not differ significantly in any demographic characteristics or in baseline balance test, musculoskeletal well-being, and muscle relaxation (Table 1). However, values of surefootedness and sense of balance were significantly higher in the control group than in the training group at day 1. Means, standard deviations, and correlations among study variables are shown in Table 2.

### 3.1. Daily Measurements of Surefootedness and Sense of Balance and Musculoskeletal Well-Being and Muscle Relaxation over Time

**Figure 3:**
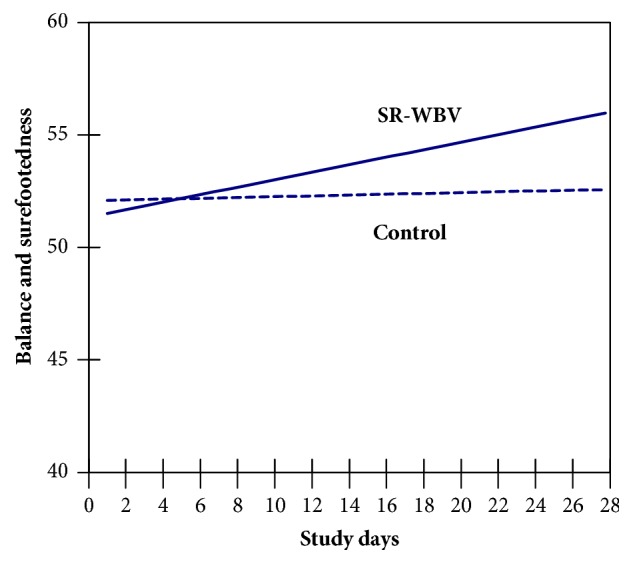
Effect of SR-WBV condition on daily surefootedness and sense of balance across 28-day study period.

**Figure 4:**
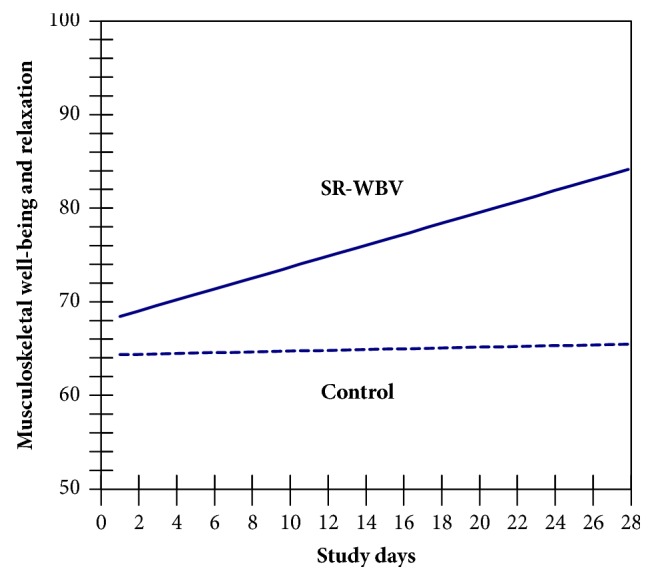
Effect of SR-WBV condition on daily musculoskeletal well-being and muscle relaxation across 28-day study period.

Multilevel results for surefootedness and sense of balance, as well as for musculoskeletal well-being and muscle relaxation, are listed in Table 3. Across study days SR-WBV had a significant influence on surefootedness and sense of balance (B = 0.142, SE = 0.057, t = 2.491, df = 55, t_krit_ = 2.004, p = .016, and two-tailed) as shown in a significant interaction between days of study and SR-WBV exercise (Figure 3). The interaction was also significant in prediction of musculoskeletal well-being and muscle relaxation (B = 0.528, SE = 0.208, t = 2.538, df = 55, t_krit_ = 2.004, p = .014, and two-tailed; see Figure 4). These results support our hypotheses 1 and 3.

### 3.2. Balance Tests

**Figure 5:**
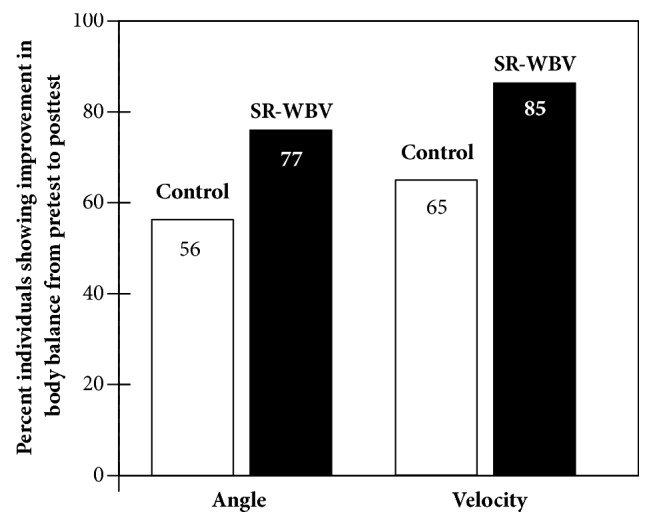
Improvement in body balance tests by SR-WBV condition.

Figure 5 shows the frequency of improvement in body balance for intervention groups (individual pre- minus postintervention trunk sway values). In the control group improvement was not significant (did not differ from 50% chance expectation) in TotAngArea (Z = 0.514, p = .607) and TotVelArea (Z = 1.886, p = .059). However, in the SR-WBV training group there were more frequent improvements that differed from chance expectation in TotAngArea (Z = 2.550, p = .011) and TotVelArea (Z = 3.334, p = .001). As lower values in TotAngArea as well as in TotVelArea stand for a better balance, these results support our hypothesis 2.

## 4. Discussion

SR-WBV is an upcoming health intervention for the occupational setting. So far, promising effects were shown in metal-manufacturing workers [23], health-care professionals, and nurses [21, 35]. To our knowledge there is only one study focusing on the effects of SR-WBV on balance in office workers [18, 22] that found an improvement of balance after four weeks of SR-WBV. As that result, due to restrictions of spatial arrangements, was not compared with a control group, studies were needed that pay particular attention to the effects of SR-WBV on balance compared to a control group. Kaeding et al. [22] implemented a 3-month sinusoidal WBV intervention in office workers comparing training and control group but found no improvement of balance. As we found an improvement of balance after a shorter intervention of 4 weeks SR-WBV, which was not found after 3 months of sinusoidal WBV, it might underline the special nature of stochastic vibration that acts as an exercise for the sensorimotor system [25]. As described by Maki et al. [9], an increased balance may reduce the risk of falls. Compared to conventional balance trainings, SR-WBV exercise has many advantages as no warm-up, cool down, or training clothes are needed [35]. As SR-WBV exercise takes only a short time and no effort from participants, compliance rates for SR-WBV exercises are higher than with other worksite activity program [19]. Therefore, it seems ideal for the working context. Especially office workers who spend most of their time in sitting position could benefit from SR-WBV exercise to improve balance and reduce the risk of falls. Compliance rates in this study were high and daily questionnaires showed that surefootedness and sense of balance improved over time in those who did SR-WBV but not in those who were not doing the SR-WBV exercise. Also, musculoskeletal well-being and muscle relaxation improved over time in those who did the SR-WBV exercise but not in those who did no SR-WBV exercise. We replicated the findings of Burger et al. [23], as we used SR-WBV on a relatively healthy sample of office workers. A limitation of this study is that there was no placebo intervention. Due to a low number of participants, this second control group was not feasible. Therefore, a weakness of this study is the small sample size. Also, all participants attended voluntarily and were not blind with regard to their training or control group assignment. Because of that, results may be influenced from participants beliefs about the effectiveness of SR-WBV. As organizations may especially be interested in the lasting effects of SR-WBV exercise, further research may also include follow-up measurements after several months. Another limitation of this study is related to the relatively young age of participants. Since falls and slips are particularly common in the elderly further studies with older people are needed.

## 5. Conclusions

In this longitudinal randomized-controlled study we could show that surefootedness and sense of balance, as well as musculoskeletal well-being and muscle relaxation, increased during a 4-week SR-WBV intervention with office workers. In addition to these self-report measurements, performance on a balance test increased significantly after the SR-WBV intervention, whereas those who received no treatment showed no improvement. Especially office workers who spend most of their time in sitting position could benefit from SR-WBV exercise to improve balance and reduce the risk of falls. Further research should focus on older people, since falls and slips are more common in the elderly. Future studies should also include follow-up measurements to test the lasting effects of SR-WBV exercise.

## Figures and Tables

**Table 1:** Descriptive and inferential statistics of study variables at baseline.

	**Training group** **(n = 26)**		**Control group** **(n = 36)**			
**Variable**	**M**	**SD**	**M**	**SD**	***t***	***P***
**Sex**	15 m; 11 f		17 m; 19 f			
**Smoking**	4 s; 22 ns		6 s; 30 ns			
**Age (y)**	36.23	14.46	43.33	13.37	-1.995	0.051
**BMI (kg/m** ^**2**^ **)**	24.10	3.67	25.27	3.82	-1.174	0.245
Health Status	4.19	0.75	4.09	0.75	0.531	0.597
Sport Activity	3.88	1.58	3.85	1.26	0.086	0.931
Balance-Test 1 (TotAngArea)	45.81	50.91	66.87	94.28	-1.034	0.305
Balance-Test 1 (TotVelArea)	643.73	652.91	806.09	1117.82	-0.663	0.510
Surefootedness (Day 1)	47.85	5.76	52.64	8.90	-4.792	0.019
Sense of balance (Day 1)	47.38	6.52	51.86	8.86	-4.477	0.033
MSK Well-being (Day 1)	68.77	33.35	73.81	26.48	-0.663	0.510
Muscle Relaxation (Day 1)	65.85	34.31	69.83	26.64	-0.515	0.608

Health Status was assessed from 1 very bad to 5 very good; Frequency of Sport Activity was assessed from 1 never to 7 several times a day; Outcomes of the Balance-Test 1 were Total Angle Area (TotAngArea) and Total Velocity Area (TotVelArea) whereas a lower value means better balance; Surefootedness, Sense of balance, Musculoskeletal (MSK) Well-being and Muscle Relaxation on Day 1.

**Table 2:** Means, standard deviations, and correlations among study variables.

	*Means *±SD	Sure-footedness	Sense of Balance	MSK Well-being	Muscle Relaxation	Balance-Test 1 (Angle)	Balance-Test 2 (Angle)	Balance-Test 1 (Velocity)	Balance-Test 2 (Velocity)
(1) Surefootedness	51.13±6.18	1							
(2) Sense of Balance	51.05±6.18	.883^∗∗^	1						
(3) Musculoskeletal Well-being	78.25±25.93	.057	.080^∗∗^	1					
(4) Muscle Relaxation	77.43±26.19	.063^∗^	.091^∗∗^	.962^∗∗^	1				
(5) Balance-Test 1 (Angle)	64.13±85.71	.096^∗∗^	.063^∗^	-.148^∗∗^	-.115^∗∗^	1			
(6) Balance-Test 2 (Angle)	57.14±89.72	-.024	-.022	-.188^∗∗^	-.139^∗∗^	.333^∗∗^	1		
(7) Balance-Test 1 (Velocity)	810.05±1031.27	.086^∗∗^	.052	-.150^∗∗^	-.126^∗∗^	.956^∗∗^	.221^∗∗^	1	
(8) Balance-Test 2 (Velocity)	623.55±852.18	.003	.003	-.204^∗∗^	-.143^∗∗^	.411^∗∗^	.942^∗∗^	.310^∗∗^	1
(9) Sport Activity	3.85±1.32	.068^∗^	.068^∗^	.091^∗∗^	.093^∗∗^	-.081^∗∗^	.034	-.150^∗∗^	.094^∗∗^
(10) Age	40.60±13.91	.105^∗∗^	.086^∗∗^	-.036	-.001	.363^∗∗^	.224^∗∗^	.351^∗∗^	.258^∗∗^
(11) BMI	24.73±3.59	.150^∗∗^	.121^∗∗^	-.185^∗∗^	-.165^∗∗^	.081^∗∗^	.039	.058^∗^	.057
(12) Sex	1.49±0.50	-.135^∗∗^	-.142^∗∗^	.077^∗∗^	.060^∗^	.066^∗^	.117^∗∗^	.047	.045

*Note. N* = 1165 daily reports from 62 participants. ^*∗*^*p* < 0.05, ^*∗∗*^*p* < 0.01, ^*∗∗∗*^*p* < 0.001, and two-tailed.

**Table 3:** Prediction of daily surefootedness, sense of balance, musculoskeletal well-being, and muscle relaxation in multilevel regression analyses.

	Musculoskeletal well-being & muscle relaxation		Surefootedness and sense of balance	
	B	SE	B	SE
Constant	64.310	9.184	52.072	1.807
Sex	5.669	5.467	-1.019	1.059
BMI	-1.940^∗^	0.735	0.104	0.143
Age	0.391	0.203	0.018	0.039
SR-WBV	3.574	5.713	-0.724	1.219
Day	0.020	0.133	0.017	0.037
Interaction Effects				
Day X SR-WBV	0.519^∗^	0.206	0.142^∗^	0.057
Level 2 variance	325.959	64.043	11.246	2.386
Level 1 variance	283.758	13.720	22.398	1.082
IGLS	7931.824		5564.738	

*Note. n* = 915 daily ratings reported by 61 participants across 28 days. B = fixed parameter estimates of unstandardized regression coefficients; *SE* = standard error in unstandardized regression coefficients estimation; significance levels were calculated by t-values (parameter estimate/SE) with j-p-1 degrees of freedom, where j is the number of units on level 2 and p is the number of explanatory variables. Sex (1 = m, 2 = f); SR-WBV = stochastic resonance whole-body vibration training (0 = control group, 1 = training group); day = day of training period (1-28).

^*∗*^ *p* < 0.05, ^*∗∗*^*p* < 0.01, ^*∗∗∗*^*p* < 0.001, and two-tailed.

## Data Availability

The data used to support the findings of this study are available upon request.

## References

[1] Unfallversicherung K. F. D. S. D., SUVA (2017). Unfallstatistik UVG 2017. *Koordinationsgruppe für die Statistik der Unfallversicherung*.

[2] EKAS Arbeitssicherheit und Gesundheitsschutz in Bürobetrieben. http://www.ekas.admin.ch/redirect.php?cat=f%2BR3rLeQL4g%3D&id=94.

[3] Collins J. W., Bell J. L., Grönqvist R. Multidisciplinary research to prevent SLIP, TRIP, and FALL (STF) incidents among hospital workers.

[4] Burdorf A., Koppelaar E., Evanoff B. (2013). Assessment of the impact of lifting device use on low back pain and musculoskeletal injury claims among nurses. *Occupational and Environmental Medicine*.

[5] Gauchard G. C., Chau N., Touron C. (2003). Individual characteristics in occupational accidents due to imbalance: A case-control study of the employees of a railway company. *Occupational and Environmental Medicine*.

[6] Kemmlert K., Lundholm L. (2001). Slips, trips and falls in different work groups - With reference to age and from a preventive perspective. *Applied Ergonomics*.

[7] Era P., Sainio P., Koskinen S., Haavisto P., Vaara M., Aromaa A. (2006). Postural balance in a random sample of 7,979 subjects aged 30 years and over. *Gerontology*.

[8] SUVA Geschäftsbericht 2012. https://www.suva.ch/material/dokumentationen/geschaeftsbericht%202012%20vollversion.

[9] Maki B. E., Sibley K. M., Jaglal S. B. (2011). Reducing fall risk by improving balance control: Development, evaluation and knowledge-translation of new approaches. *Journal of Safety Research*.

[10] World Health Organization Physical activity strategy for the WHO European Region 2016–2025. http://www.euro.who.int/en/publications/abstracts/physical-activity-strategy-for-the-who-european-region-20162025.

[11] Patti A., Bianco A., Karsten B. (2017). The effects of physical training without equipment on pain perception and balance in the elderly: A randomized controlled trial. *Work*.

[12] Lechner L., De Vries H., Adriaansen S., Drabbels L. (1997). Effects of an employee fitness program on reduced absenteeism. *Journal of Occupational and Environmental Medicine*.

[13] Robroek S. J. W., van Lenthe F. J., van Empelen P., Burdorf A. (2009). Determinants of participation in worksite health promotion programmes: a systematic review. *International Journal of Behavioral Nutrition and Physical Activity*.

[14] Kaewthummanukul T., Brown K. C. (2006). Determinants of employee participation in physical activity: critical review of the literature. *AAOHN Journal*.

[15] Elfering A., Mannion A. F. (2008). Epidemiology and risk factors of spinal disorders. *Spinal Disorders*.

[16] Rogan S., Hilfiker R., Herren K., Radlinger L., de Bruin E. D. (2011). Effects of whole-body vibration on postural control in elderly: a systematic review and meta-analysis. *BMC Geriatrics*.

[17] Kaeding T. S. (2017). Whole-body vibration training in workplace-health promotion: a promising intervention?. *Austin Sports Medicine*.

[18] Elfering A., Arnold S., Schade V., Burger C., Radlinger L. (2013). Stochastic resonance whole-body vibration, musculoskeletal symptoms, and body balance: A worksite training study. *Safety and Health at Work*.

[19] Awan R. I., khan N., Perveen S. (2017). The effect of WBV on balance, mobility and strength in aging adults: a systematic review. *Biological Systems: Open Access*.

[20] Del Pozo-Cruz B., Hernández Mocholí M. A., Adsuar J. C., Parraca J. A., Muro I., Gusi N. (2011). Effects of whole body vibration therapy on main outcome measures for chronic non-specific low back pain: a single-blind randomized controlled trial. *Journal of Rehabilitation Medicine*.

[21] Elfering A., Duffy V., Lightner N. (2014). Stochastic resonance training at work reduces musculoskeletal pain in nurses. *Advances in Human Aspects of Healthcare*.

[22] Kaeding T. (2027). Whole-body vibration training as a workplace-based sports activity for employees with chronic low-back pain. *Scandinavian Journal of Medicine & Science in Sports*.

[23] Burger C. (2012). Stochastic resonance training reduces musculoskeletal symptoms in metal manufacturing workers: A controlled preventive intervention study. *Work*.

[24] Ward L. M., Neiman A., Moss F. (2002). Stochastic resonance in psychophysics and in animal behavior. *Biological Cybernetics*.

[25] Haas C. T. (2006). The effects of random whole-body-vibration on motor symptoms in Parkinson's disease. *NeuroRehabilitation*.

[26] Luginbuehl H., Lehmann C., Gerber R. (2012). Continuous versus intermittent stochastic resonance whole body vibration and its effect on pelvic floor muscle activity. *Neurourology and Urodynamics*.

[27] Ross S. E., Arnold B. L., Blackburn J. T., Brown C. N., Guskiewicz K. M. (2007). Enhanced balance associated with coordination training with stochastic resonance stimulation in subjects with functional ankle instability: An experimental trial. *Journal of NeuroEngineering and Rehabilitation*.

[28] Schollhorn W. I. (2006). Does noise provide a basis for the unification of motor learning theories?. *International Journal of Sport Psychology*.

[29] Bell D. R., Guskiewicz K. M., Clark M. A., Padua D. A. (2011). Systematic review of the balance error scoring system. *Sports Health: A Multidisciplinary Approach*.

[30] Toigo M., Boutellier U. (2006). New fundamental resistance exercise determinants of molecular and cellular muscle adaptations. *European Journal of Applied Physiology*.

[31] Portney L., Watkins M. (2009). *Foundations of Clinical Research: Applications to Practice*.

[32] Singer J. D., Willett J. B. (2003). Applied longitudinal data analysis: Modeling change and event occurrence. *Applied Longitudinal Data Analysis: Modeling Change and Event Occurrence*.

[33] Rasbash J. (2017). *A User’s Guide to MLwiN*.

[34] IBM_Corp R. IBM SPSS Statistics for Macintosh.

[35] Elfering A. (2014). Stochastic resonance whole-body vibration improves postural control in health care professionals: a worksite randomized controlled trial. *Workplace Health & Safety*.

